# Simultaneous detection and ribotyping of *Clostridioides difficile*, and toxin gene detection directly on fecal samples

**DOI:** 10.1186/s13756-020-00881-9

**Published:** 2021-01-29

**Authors:** Tessel M. van Rossen, Joffrey van Prehn, Alex Koek, Marcel Jonges, Robin van Houdt, Rosa van Mansfeld, Ed J. Kuijper, Christina M. J. E. Vandenbroucke-Grauls, Andries E. Budding

**Affiliations:** 1 Department of Medical Microbiology and Infection Control, Amsterdam Infection and Immunity Institute, Amsterdam UMC, Vrije Universiteit Amsterdam, PK 2X132, De Boelelaan 1117, Amsterdam, The Netherlands; 2 Center for Infectious Diseases, Department of Medical Microbiology, Leiden University Medical Center, Albinusdreef 2, Leiden, The Netherlands; 3 inBiome B.V., Science park 106, Amsterdam, The Netherlands

**Keywords:** *Clostridioides difficile*, PCR ribotyping, Toxin genes, Infection control, Hospital epidemiology

## Abstract

**Background:**

*Clostridioides difficile* is the most common cause of nosocomial diarrhea. Ribotyping of cultured strains by a PCR-based test is used to study potential transmission between patients. We aimed to develop a rapid test that can be applied directly on fecal samples for simultaneous detection and ribotyping of *C. difficile*, as well as detection of toxin genes.

**Methods:**

We developed a highly specific and sensitive primer set for simultaneous detection and ribotyping of *C. difficile* directly on total fecal DNA. Toxin genes were detected with primers adapted from Persson et al. (Clin Microbiol Infect 14(11):1057–1064). Our study set comprised 130 fecal samples: 65 samples with positive qPCR for *C. difficile* toxin A/B genes and 65 *C. difficile* qPCR negative samples. PCR products were analyzed by capillary gel electrophoresis.

**Results:**

Ribosomal DNA fragment peak profiles and toxin genes were detected in all 65 *C. difficile* positive fecal samples and in none of the 65 *C. difficile* negative samples. The 65 samples were assigned to 27 ribotypes by the Dutch reference laboratory. Our peak profiles corresponded to these ribotypes, except for two samples. During a *C. difficile* outbreak, patients were correctly allocated to the outbreak-cluster based on the results of direct fecal ribotyping, before *C. difficile* isolates were cultured and conventionally typed.

**Conclusion:**

*C. difficile* ribotyping directly on fecal DNA is feasible, with sensitivity and specificity comparable to that of diagnostic toxin gene qPCR and with ribotype assignment similar to that obtained by conventional typing on DNA from cultured isolates. This supports simultaneous diagnosis and typing to recognize an outbreak.

## Background

*Clostridioides difficile* is an anaerobic, spore-forming bacterium and the most common cause of hospital-associated diarrhea. In severe cases, *C. difficile* infection (CDI) can lead to pseudomembranous colitis, toxic megacolon and bowel perforation. The attributable mortality of *C. difficile* infection in an endemic setting is approximately 5% [1, 2]. Hospital outbreaks of *C. difficile* occur often, presumably due to the large numbers of bacterial spores that can be excreted by symptomatic patients. Outbreaks threaten patient safety, and pose a substantial financial burden to healthcare. Incremental costs per CDI are estimated to be approximately €5000 ($5700), but in outbreak-settings these can increase to €7000–16,000 ($7.900–18.100) per patient [3, 4].

To detect *C. difficile* transmission between patients, bacterial strains need to be characterized beyond the species level. Furthermore, early recognition of hypervirulent strains is important for prompting institution of strict infection control measures, since these strains are associated with higher mortality and transmission rates [5–7]. A commonly used typing technique for *C. difficile*, which is recommended for surveillance purposes, is PCR ribotyping [8]. This method is based on strain-specific differences in number and length of the ribosomal 16-23S interspace regions (IS-regions). A drawback of the currently used PCR ribotyping methods is that they can only be performed on cultured *C. difficile* isolates [9–14]. This delays the time to results and eventual implementation of infection control measures. Therefore, Janezic et al. designed new primers and tested these directly on total fecal DNA; they obtained a specificity of 100% and a sensitivity of 84.8% [15].

Our objective was to develop ribotyping primers which could also be applied directly on fecal DNA but with higher sensitivity, while retaining specificity. Ideally, direct ribotyping on feces should be as sensitive as *C. difficile* quantitative PCR (qPCR), as this would make it possible to use it as first-line diagnostic assay. We assessed this new method during a suspected outbreak of *C. difficile* in our hospital. Thereafter, we validated our primers and our optimized protocol in a larger sample set of *C. difficile*-positive and -negative stools to assess sensitivity, specificity, and typing performance.

## Methods

### Primers

Primers were designed with AliView (Uppsala University, Uppsala, Sweden) based on alignment of 20 downloaded *C. difficile* sequences from the Silva database (Max Planck Institute for Marine Microbiology and Jacobs University, Bremen, Germany) [16]. Specificity was assessed by comparison to *C. difficile* closest phylogenetic relatives, *C. sordellii* and *C. bifermentans*. Primers were targeted to the 16S-23S ribosomal DNA interspace regions (IS-regions). Since we aimed to perform ribotyping directly in fecal samples comprising high loads of non-*C. difficile* bacteria, we attempted to improve specificity for *C. difficile* by shifting the primers from the more conserved 16S region towards the IS-region. We observed that different primers were needed for amplification of short (< 400 nucleotides) and long (> 400 nucleotides) IS-fragments. This resulted in the four primers shown in Additional file 1: Table 1. Using BLAST, we observed a 100%/100% match with the 20 *C. difficile* sequences and no cross reactivity with *C. sordellii* and *C. bifermentans*, which are taxonomically closest to *C. difficile*. Forward ribotyping primers were FAM-labeled for analysis with ABI Prism 3500 GeneticAnalyzer (Applied Biosystems, Foster City, California, USA). For detection of toxin A (*tcdA*), toxin B (*tcdB*), binary toxin (*cdtA*, *cdtB*) genes, we used the primers designed by Persson et al. (Additional file 1: Table 1) [17]. Forward toxin gene primers were HEX-labeled.

### Fecal samples and *C. difficile* strains

During the optimization phase of our direct ribotyping technique, a *C. difficile* outbreak was suspected in the Intensive Care Unit (ICU) of our institution. To assess the clinical applicability of our method, we applied this new technique directly to the fecal samples of eleven patients with positive *C. difficile* tests. Thereafter, we validated our method in a larger study set of 130 fecal samples: in addition to the eleven samples from the outbreak, we randomly selected 54 fecal samples with positive qPCR for *C. difficile* toxin A and/or B genes (the standard diagnostic test for *C. difficile* detection in our laboratory) and 65 *C. difficile* qPCR negative samples, derived from the diagnostic laboratory. For control, *C. difficile* strains were cultured from all 65 fecal samples with positive qPCR for *C. difficile* toxin A and/or B genes. Culture was performed anaerobically on selective *C. difficile* agar plates (bioMérieux, Marcy l'Etoile, France) according to standard protocol of our diagnostic microbiological laboratory. All 65 *C. difficile* strains were also sent to the Dutch National Reference Laboratory at Leiden University Medical Center (LUMC) for conventional ribotyping using a standardized protocol [18]. These ribotypes served as reference. As control samples we randomly selected 65 fecal samples with negative qPCR’s for *C. difficile* from the routine diagnostic microbiology laboratory. Of these *C. difficile* negative samples, three were positive in PCR for *Salmonella* spp., six for *Campylobacter* spp., one for *Shigella* spp., one for both *Campylobacter* spp. and *Shigella* spp., one for parechovirus, one for norovirus and one for enterovirus. To assess potential cross-reactivity in vitro, we also performed direct ribotyping on *C. difficile’s* closest taxonomically relatives, *C. sordellii* and *C. bifermentans*. These isolates were derived from the diagnostic laboratory and identified with the MALDI-TOF MS (Matrix-Assisted Laser Desorption—Ionisation-Time of Flight Mass Spectrometry, VITEK MS, BioMerieux). All fecal samples were obtained from hospitalized patients with diarrhea, admitted to Amsterdam UMC, location VUmc, between 2016 and 2018 (Additional file 1: Table 2).

### DNA isolation from fecal samples and *C. difficile* strains

DNA isolation was performed according to standard protocol of our diagnostic microbiological laboratory. Within 36 h after arrival at the laboratory, fecal samples were stored at − 80 °C. For this study, samples were thawed and a pea-sized amount of feces (100–400 mg) was collected with a swab. In case of liquid feces, swabs were immersed halfway into the liquid. Swabs were placed in Eppendorf tubes, vortexed and incubated in 1 ml S.T.A.R. buffer (Roche, Basel, Switzerland) at − 80 °C for 1 h or overnight. Subsequently, tubes were heated at 100 °C for 10 min. After centrifugation for 10 s at 4000 rpm, 300 µl of the supernatant fraction was suspended in 300 µl lysis buffer for DNA isolation with MagNaPure96 (Roche, Basel, Switzerland). *C. difficile* strains were collected with a 1 µl inoculation loop and stored in 200 µl TE-lysis buffer (Tris–HCl, EDTA, pH 8.0, Promega V6231) at − 20 °C. After thawing, suspensions were vortexed for 15 s and centrifuged for 3 min at 12,000 rpm. The supernatant was diluted 1:10 (1 µl supernatant and 9 µl nuclease free water) and 15 µl Mastermix was added for the PCR reaction.

### Amplification and analysis

For both PCR reactions of direct ribotyping and toxin gene detection, extracted DNA of cultured strains was diluted 1:10; DNA of fecal samples was used undiluted. When inhibition of the PCR reaction was suspected (no peaks or primer-dimer signal detected), the reaction was repeated with total fecal DNA diluted 1:5 to identify a possible false negative result due to inhibition. PCR mixtures for ribotyping, with a final volume of 25 µl consisted of 10 µl DNA and 15 µl of IS-pro mastermix (inBiome bv) with 0.13 µM of each primer. PCR mixtures for toxin gene detection, with a final volume of 25 µl consisted of 10 µl DNA and 15 µl IS-pro mastermix (inBiome bv) with 0.6 µM of each *tcdA*-primer, 0.4 µM *tcdB*-F primer, 0.2 µM of each *tcdB*-R primer, 0.05 of each *cdtA*-F primer, 0.1 µM *cdtA*-R primer and 0.1 µM of each *cdtB*-primer. PCR mixtures for ribotyping and toxin gene detection were placed in separate wells. Amplifications were carried out with GeneAmp PCR system 9700 (Applied Biosystems, Foster City, CA, USA). Cycling conditions for both ribotyping and toxin gene detection PCRs were 95 °C for 10 min, 12 cycles (with 0.7 °C decrements at every cycle) of 94 °C for 30 s, 65 °C for 30 s and 72 °C for 1 min and 23 cycles of 94 °C for 30 s, 56 °C for 30 s and 72 °C for 1 min, followed by extension at 72 °C for 11 min. Afterwards PCR product was stored at 4 °C. Within 12 h, 5 µl PCR product and 20 µl formamide with custom size marker (eMix, InBiome, Amsterdam, the Netherlands) was pipetted in a 96-wells plate, heated at 94 °C for 3 min and cooled down to 4 °C. DNA fragment analysis was performed with ABI Prism 3500 GeneticAnalyzer (Applied Biosystems, Foster City, California, USA) in separate capillaries for direct ribotyping (FAM-labeled primers) and toxin gene detection (HEX-labeled primers). DNA fragment lengths including intensity were visualized and analyzed with TIBCO Spotfire (TIBCO Software Inc., Palo Alto, California, USA). To standardize the amount of bacterial DNA, we calculated relative intensity of each ribosomal DNA fragment peak by dividing the absolute intensity of each peak of a sample by the absolute intensity of the highest peak of that sample. Clustering of fecal samples based on ribotype DNA fragment peak profile similarity was performed by UPGMA (Unweighted Pair Group Method with Arithmetic Mean), with cosine correlation as distance measure and average value as ordering weight. Toxin gene-specific peaks were defined according to DNA fragment sizes described by Persson et al.: *tcdA* (629 bp), *tcdB* (410 bp), *cdtA* (221 bp) and *cdtB* (262 bp). Presence or absence of toxin gene peaks was scored binarily using an intensity cutoff of 3000 RFU.

## Results

### Application of direct ribotyping during an outbreak with *C. difficile*

**Fig. 1:**
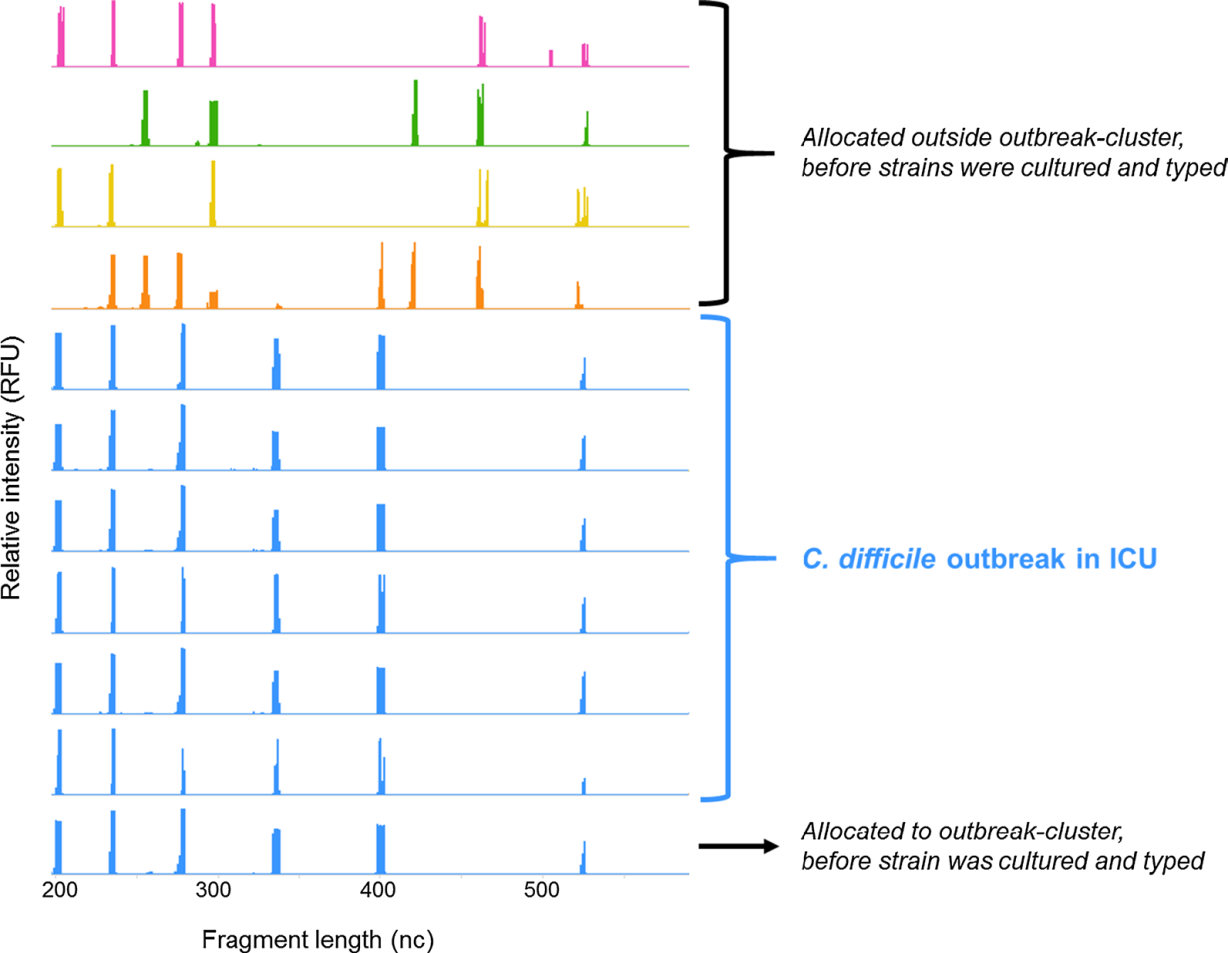
Ribosomal DNA fragment profiles in fecal samples of eleven patients with positive *C. difficile* toxin A and/or B genes qPCR. Bacterial transmission was suspected in seven patients with identical peak profiles (blue). nc = nucleotides, RFU = relative fluorescence units

During the optimization phase of our direct ribotyping technique, a *C. difficile* outbreak was suspected in the ICU. In our institution, the standard typing technique for *C. difficile* is Amplified Fragment Length Polymorphism (AFLP) on cultured strains. The suspected outbreak cluster involved six patients with the same *C. difficile* AFLP-type*.* During this outbreak, samples of five other patients became positive for *C. difficile* by qPCR or toxin enzyme immune assay (EIA). We performed direct ribotyping on total fecal DNA of all eleven patients. The six patients with the same *C. difficile* AFLP-type had identical ribotype peak profiles (Fig. 1). In the five other patients that became positive for *C. difficile* during the outbreak, direct ribotyping enabled us to allocate 1 of the 5 patients to the outbreak-cluster and 4/5 patients outside the outbreak-cluster (Fig. 1). Importantly, results of direct fecal ribotyping were obtained before strains were cultured and conventionally typed by AFLP.

### *C. difficile* PCR ribotyping and toxin gene detection

After our first experience with direct ribotyping during the outbreak, we aimed to validate our technique with a larger sample set of 130 fecal samples (including the 11 samples collected during the outbreak). With the ribotype primers we amplified DNA of a total of 65 fecal samples that were previously proven to contain *C. difficile* by qPCR for *C. difficile* toxin A and/or B genes (the standard diagnostic test for *C. difficile* detection in our laboratory). Mean Cp value (crossing point at which the amplification curve crosses the vertical threshold line; this is inversely associated with the *C. difficile* load) of *C. difficile* toxin gene qPCR was 33 (range 27–40 cycles, Additional file 1: Table 2). DNA fragment peak profiles were obtained from all 65 fecal samples (3 after 1:5 dilution because of inhibition) and from all 65 cultured strains. Hence, the sensitivity of the new primers set for toxigenic *C. difficile* detection was 100% (n = 65, 95% Confidence Interval (CI) 94.5–100%).

We observed DNA fragment peaks ranging in size from approximately 200 to 590 nucleotides, consistent with published studies when corrected for differences in primer binding sites [11, 12, 14, 15]. Also the number of DNA fragments was in agreement with previously described ribotype profiles, and varied between 5 and 10 [11, 12, 14, 15].

To examine the specificity of our primers for *C. difficile* detection, we applied the primers to total DNA obtained from *C. bifermentans* and *C. sordellii* strains and 65 fecal samples with negative qPCR for *C. difficile* toxin genes. Of these samples, fourteen were positive by diagnostic PCR’s for other bacterial species and viruses that are well-known causes of diarrhea such as *Campylobacter spp.*, *Salmonella spp.* and norovirus. No DNA fragment peak profiles were detected in these samples, indicating a diagnostic specificity of 100% (n = 65, 95% CI 94.5–100%).

To assess reproducibility, DNA isolation and direct ribotyping was performed *in duplicate* on a subset of 40 fecal samples with a positive qPCR for *C. difficile* toxin A and/or B genes. DNA fragment peak profiles were observed in 40/40 paired fecal samples. Profiles of 36/40 paired fecal samples were 100% identical (90%). All discrepancies were found in larger DNA fragments (> 400) in low load samples (*C. difficile* toxin A and/or B genes qPCR Cp values 35–39).

**Fig. 2:**
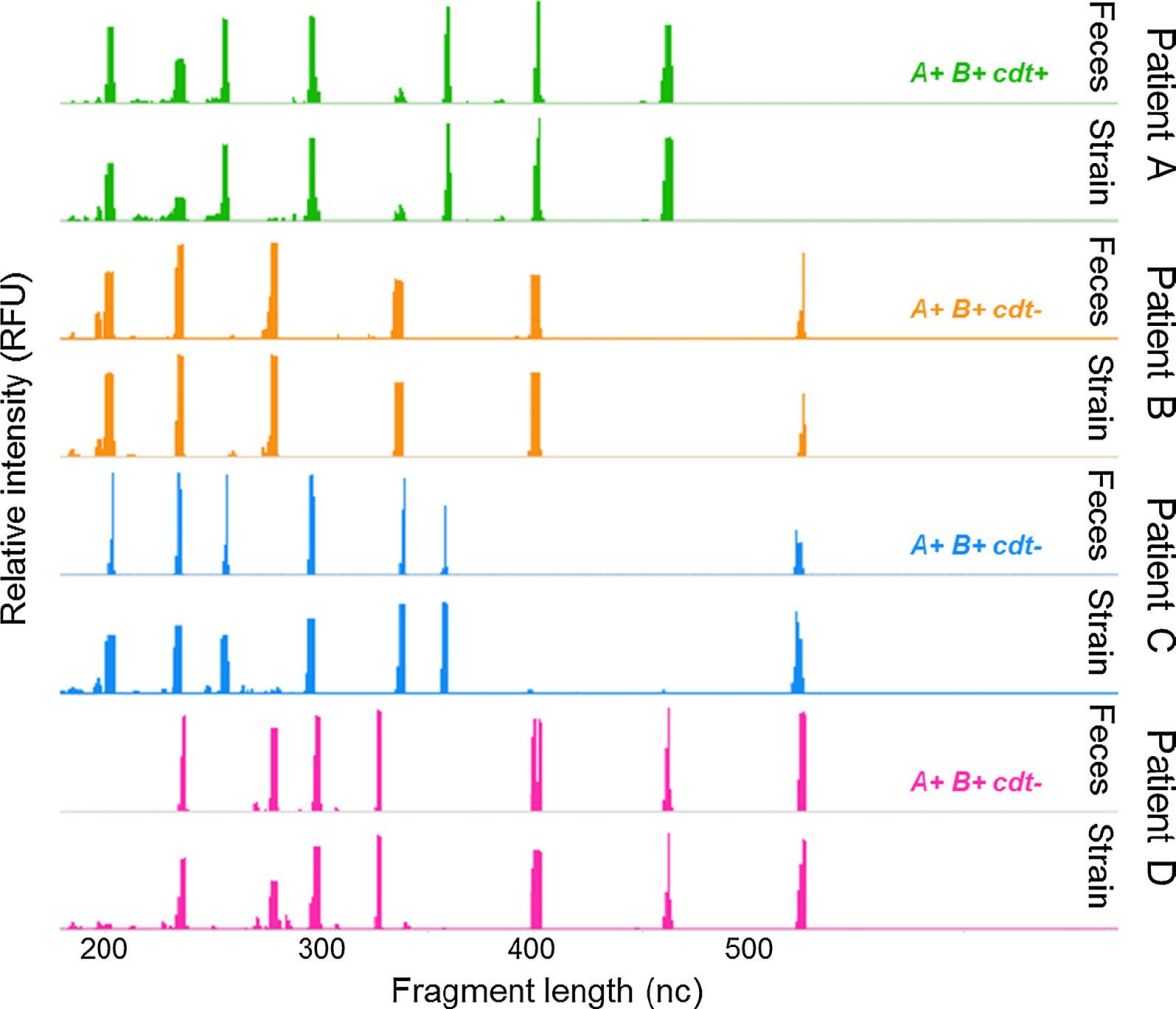
Examples of DNA fragment peak profiles from four fecal samples and their corresponding cultured strains. nc = nucleotides, RFU = relative fluorescence units, A = toxin A gene, B = toxin B gene, cdt = binary toxin genes.

To examine possible technical issues of ribotyping directly on feces—for example decreased intensity of DNA fragment peaks due to PCR inhibition or appearance of nonspecific peaks due to an excess of fecal DNA—the peak profile of each fecal sample was compared with that of its corresponding cultured strain, see Fig. 2 for example. Peak profiles of 61/65 paired fecal samples and strains were completely identical (94%). In 3/65 samples we observed 1 peak difference. These samples had a low bacterial load in qPCR (Cp values 35–39); and it was one of the larger DNA fragment peaks (> 400 nucleotides) that was missing. In 1/65 samples we observed that the three largest DNA fragments in the strain profile were missing in the profile of the fecal sample (Cp value 29).

**Fig. 3:**
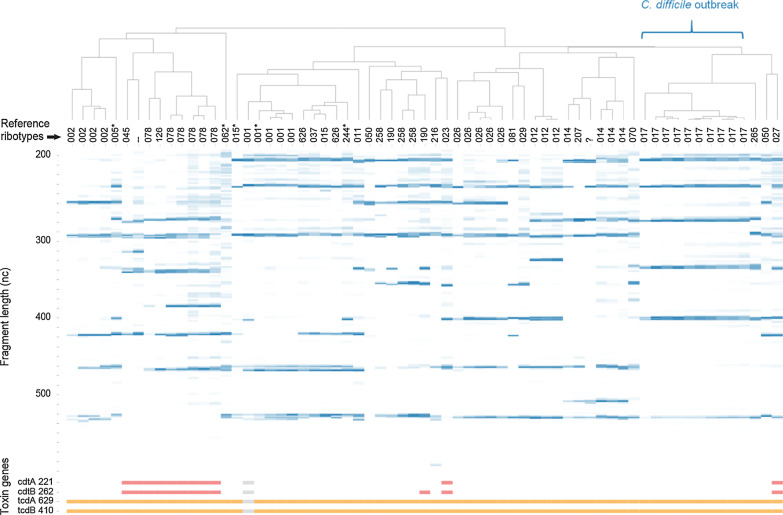
Heat map and dendrogram based on ribosomal DNA fragment peak profiles of all fecal samples. Each column represents one sample. Numbers on the X-axis correspond to the reference ribotype assigned to the corresponding strain by conventional ribotyping by the Dutch National Reference Laboratory. Numbers on the Y-axis correspond to the DNA fragment length of the detected ribotype DNA fragment peaks/bands (in blue) and/or *C. difficile* toxin genes (red/orange) per sample. Toxin gene detection could not be performed in one sample due to insufficient DNA material (grey). ‘*’ = probable reference ribotype (by conventional ribotyping by the Reference Laboratory, band patterns of study strains were highly similar to patterns of reference strains except for a 1 or 2 bands difference); ‘?’ = unknown reference ribotype (by conventional ribotyping, an unknown band pattern was observed in the study strain that did not match the band pattern of one of the reference strains); ‘-’ = no reference ribotype (by conventional ribotyping, no band pattern was observed and therefore this study strain could not be matched to a reference ribotype); tcdA = *C. difficile* toxin A; tcdB = *C. difficile* toxin B; cdtA = *C. difficile* binary toxin A; cdtB = *C. difficile* binary toxin B, nc = nucleotides

For detection of toxin A (*tcdA*), toxin B (*tcdB*) and binary toxin (*cdtA*, *cdtB*) genes directly on total fecal DNA we used primers designed by Persson et al. and added these in our study set (Fig. 3) [17]. All *C. difficile* positive fecal samples showed at least one toxin gene peak, whereas no peaks were observed in the *C. difficile* negative fecal samples. The presence of toxin genes specific for different ribotypes was consistent with literature [11, 19–21]. In one sample with RT190, toxin A, B and binary toxin B genes were detected but not binary toxin A gene. This could be due to non-specificity of our assay; however, *C. difficile* strains with presence of binary toxin B but not binary toxin A gene have been described [22, 23]. Also, we detected both toxin A and B gene peaks in RT017 samples, while this ribotype is known to produce only toxin B [24–26]. This was observed and clarified previously by Persson et al.: “The primers used to amplify toxin A gene are located upstream of the repetitive region in the 3′-end which, in some strains, contains various deletions that render the gene product non-detectable by EIA methods. Therefore, strains that are TcdA-negative due to 3′-end deletions are still tcdA-positive according to the present multiplex PCR.” [17].

### Reference ribotypes obtained by conventional ribotyping of strains

Conventional ribotyping of all 65 *C. difficile* strains that were cultured from the 65 fecal samples was performed by the Dutch National Reference Laboratory. These ribotyping results served as reference. The Reference Laboratory could not determine the ribotype of 2/65 strains due to unknown or absent band patterns. A ‘probable ribotype’ was determined in 5/65 strains since the band patterns of these strains were highly similar to patterns of reference strains except for a 1 or 2 bands difference. Overall, 63/65 strains of our study set were assigned to 27 different reference ribotypes.

### Clustering of fecal samples based on peak profile similarity

We assessed if direct ribotyping on fecal samples was feasible as first screening tool for detection of a clonally related *C. difficile* cluster by performing cluster analysis based on ribosomal DNA fragment profile similarity. A heat map and dendrogram were created based on peak profiles of all 65 fecal samples with positive qPCR for *C. difficile* toxin A/B genes (Fig. 2). The resulting clusters consisted of fecal samples containing the same *C. difficile* ribotypes as determined by the Reference Laboratory (for example, one cluster consisted of four fecal samples that all contained RT002), except for two samples: one with RT002 and one with RT050. The ribotyping patterns in both samples lacked the larger DNA fragment peaks when compared to profiles of samples with the same reference ribotype.

In conventional ribotyping, a pattern with a single band difference is usually considered as a different ribotype. Using this definition, we assessed the performance of direct ribotyping on feces for ribotype assignment by comparing peak profiles of samples with the same reference ribotype. We observed identical peak profiles in 43/48 (90%) fecal samples containing identical ribotypes (RT001: 4 out of 5 profiles were identical, RT002: n = 3/4, RT012: n = 3/3, RT014: n = 4/4, RT015: 2/2, RT017: n = 10/10, RT026: n = 5/5, RT050 n = 0/2, RT078: n = 5/6, RT190: n = 2/2, RT258: n = 3/3, RT626: n = 2/2).

## Discussion

We developed a highly sensitive and specific set of PCR primers for *C. difficile* ribotyping that can be applied directly on fecal samples. Samples containing identical strains clustered together based on ribotype peak profile similarity. During an outbreak of *C. difficile* RT017 in our institution, patients were correctly allocated to- or outside the outbreak-cluster before *C. difficile* isolates were cultured and conventionally typed.

To the best of our knowledge, this is the third study on *C. difficile* ribotyping directly on fecal samples. Several multiplex PCRs for the detection of RT027/NAP1 strains do exist, but these are targeted at specific RT027 gene fragments and are not suitable for distinction between multiple ribotypes. Janezic et al. were the first to describe a method for direct *C. difficile* ribotyping on feces in 2011 using agarose gel electrophoresis [15]. They detected DNA fragments in 86 out of 99 *C. difficile* positive samples, resulting in a sensitivity for *C. difficile* detection of 86.9%. Recently, another research group applied the primers of Bidet et al., which were originally developed for ribotyping on cultured strains, directly on fecal DNA [12, 27]. However, one third of stool samples required broth enrichment for 24 h before ribosomal DNA fragments could be detected. Cp values of ribotyping and toxin B gene qPCRs were significantly lower in stools in which direct ribotyping was successful, compared to enriched stools. With our primers set, we detected DNA fragments in all 65 *C. difficile* positive samples, resulting in a sensitivity of 100% (95% CI 94.5–100%) without the need of broth enrichment. The specificity of our PCR was 100% (n = 65, 95% CI 94.5–100%); this was the same specificity as obtained by Janezic et al. [15]. The specificity of the method of Lloyd et al. is unclear since they did not include *C. difficile* negative samples [27].

Previous studies showed that in 5–10% of patients with CDI, two or more *C. difficile* strains can be found in the stool of these patients [27–29]. To find evidence for a mixed infection, we compared peak profiles of paired fecal samples and strains and observed 1–3 peaks difference in 4/65 paired samples. However, 3 of these profiles showed (an) extra peak(s) in the strain sample, while only one sample had one extra peak in the fecal sample. This could be an indication of a mixed infection in 1/65 samples (1.5%), this is lower than the expected percentage of mixed infections described in the literature.

A major advantage of the technique we describe is the use of high-resolution capillary gel-based electrophoresis (CE-ribotyping) instead of the conventional agarose gel-based technique [15]. With CE-ribotyping it is possible to obtain digital data and reach high levels of discrimination and reproducibility, which improves standardization of *C. difficile* ribotyping [18, 30].

A limitation of our study is the relatively small number of samples that we tested and the relatively higher number of samples with RT017, due to an outbreak. However, our set contains all major ribotypes circulating in the Netherlands, which we consider sufficient to demonstrate that direct ribotyping on fecal material is possible and accurate [31, 32]. Another limitation is that our method does not distinguish between a *C. difficile* infection or colonization, as is the case with any qPCR for diagnosis of CDI, although cycle threshold values seem to correlate with presence of free toxin [33, 34]. Therefore, a positive *C. difficile* toxin gene PCR should be followed by a positive a toxin A/B EIA to confirm the diagnosis, as recommended by the European Society of Clinical Microbiology and Infectious Diseases (ESCMID) [35]. Furthermore, the diagnosis should always be based on laboratory tests in combination with clinical symptoms or signs of CDI.

The approach described here still shows some variation in banding patterns. In low load samples, one or two bands from longer fragments may be lost. This can be a consequence of partial inhibition of the PCR reaction. This might be caused by the fact that larger DNA fragments require a longer interaction with the polymerase and the chance of an error during the annealing phase is higher. Stool broth enrichment might be an option to increase direct ribotyping success rate in samples that were unsuccessfully ribotyped [27]. In one sample, we observed that the three largest DNA fragments in the strain profile were missing in the profile of the fecal sample. We think that partial inhibition might have occurred in this sample, since the three largest DNA fragments were lacking, and it appears that large fragments are most prone to partial inhibition of the PCR reaction. Since the Cp value was 29, the inhibition cannot be explained by a low *C. difficile* load. However, since the bands were missing in the fecal sample but not in the cultured strain, we think that the feces contained specific inhibitory substances that were not present in the cultured isolate. As current ribotyping definitions consider a single band difference as a difference in ribotype, definitive assignment to ribotypes is not feasible yet. However, by using profile-based clustering the essential information for detection of *C. difficile* outbreaks can be provided.

Currently, ribotyping is still the most frequently used typing technique for general epidemiological surveys on CDI, though whole genome (or core genome) MLST (MultiLocus Sequence Typing) is increasingly used to study transmission of *C. difficile* [36, 37]. However, most of these techniques are more costly and time-consuming. At this moment, the whole process from submitting a feces sample and determining a PCR ribotype takes approximately 6 days. Our test is a PCR that can be applied directly on total fecal DNA and provides direct information on both the presence and the type of *C. difficile*. Many local diagnostic clinical microbiological laboratories nowadays only perform fecal *C. difficile* toxin gene PCR for diagnostics and hence do not culture strains for downstream molecular analysis. Since the number of laboratories with DNA sequencing devices is increasing, our technique might also become available to many local diagnostic laboratories in the near future. *C. difficile* ribotyping directly on feces could allow accelerated screening for bacterial transmission in an outbreak setting. If more detailed typing is desired, strains can be sent to a *C. difficile* reference laboratory for conventional ribotyping or MLST.

## Conclusions

We showed that *C. difficile* ribotyping and simultaneous toxin gene detection directly on fecal samples is feasible, with equal sensitivity as qPCR. This application allows for detection of *C. difficile* infection with concomitant rapid screening for bacterial transmission between patients. This may result in more timely application of infection control measures and could therefore help in limiting *C. difficile* outbreaks.

## Supplementary Information

**Additional file 1: Table 1**. Details of primers used for *C. difficile* ribotyping and toxin gene detection directly on fecal samples. **Table 2**. Details of all 65 fecal samples with positive qPCR for *C. difficile* toxin A and/or B genes and their 65 corresponding cultured strains.

## Data Availability

Details of the sample set used in the current study are shown in Additional file 1: Table 2. The datasets used and analyzed during this study are available from the corresponding author on reasonable request.
