# Supplementary material for: Simultaneous detection and ribotyping of Clostridioides difficile, and toxin gene detection directly on fecal samples
Source: Antimicrob Resist Infect Control. 2021 Jan 29;10:23. doi: 10.1186/s13756-020-00881-9 (PMC7845108; doi:10.1186/s13756-020-00881-9)
Supplement: Supplementary file 1 — Additional file 1: Table 1. Details of primers used for C. difficile ribotyping and toxin gene detection directly on fecal samples. Table 2. Details of all 65 fecal samples with positive qPCR for C. difficile toxin A and/or B genes and their 65 corresponding cultured strains. [file 13756_2020_881_MOESM1_ESM.docx]

**Supplementary material**

| **Analysis** | **Gene target** | **Primer name** | **Sequence (5′–3′)** | **Fluorescent label** | **Primer concentration (μM)** | **Amplicon size (bp)** |
| --- | --- | --- | --- | --- | --- | --- |
| *C. difficile* ribotyping PCR | *16S-23S ribosomal DNA interspace regions* | CdiffISf1 | CTGTTTAATTTTGAGGGTTCGTTTTTACG | FAM | 0.13 | varying |
|  |  | CdiffISf2a | CCTACTGTTTAATTTTGAAAGTTCTTTACG | FAM | 0.13 |  |
|  |  | CdiffISf2b | CCTACTGTTTAATTTTGAAAGTTCTTTATG | FAM | 0.13 |  |
|  |  | ClosR | AGGCATCCGCCCTGCACCCT | - | 0.13 |  |
| *C. difficile* toxin genes PCR | *tcdA* | tcdA-F3345 | GCATGATAAGGCAACTTCAGTGGTA | HEX | 0.6 | 629 |
|  |  | tcdA-R3969 | AGTTCCTCCTGCTCCATCAAATG | - | 0.6 |  |
|  | *tcdB* | tcdB-F5670 | CCAAARTGGAGTGTTACAAACAGGTG | HEX | 0.4 | 410 |
|  |  | tcdB-R6079A | GCATTTCTCCATTCTCAGCAAAGTA | - | 0.2 |  |
|  |  | tcdB-R6079B | GCATTTCTCCGTTTTCAGCAAAGTA | - | 0.2 |  |
|  | *cdtA* | cdtA-F739A | GGGAAGCACTATATTAAAGCAGAAGC | HEX | 0.05 | 221 |
|  |  | cdtA-F739B | GGGAAACATTATATTAAAGCAGAAGC | HEX | 0.05 |  |
|  |  | cdtA-R958 | CTGGGTTAGGATTATTTACTGGACCA | - | 0.1 |  |
|  | *ctdB* | ctdB-F617 | TTGACCCAAAGTTGATGTCTGATTG | HEX | 0.1 | 262 |
|  |  | cdtB-R878 | CGGATCTCTTGCTTCAGTCTTTATAG | - | 0.1 |  |

**Supplementary Table 1** – Details of primers used for *C. difficile* ribotyping and toxin gene detection directly on fecal samples. *tcdA = C. difficile toxin A; tcdB = C. difficile toxin B; cdtA = C. difficile binary toxin A; cdtB = C. difficile binary toxin B*

| **Sample no.** | **Reference ribotype identified in cultured strain** | **Toxin genes detected  in fecal sample** | **Cp value of *C. difficile* toxin A and/or B genes qPCR on fecal sample** |
| --- | --- | --- | --- |
| 1 | 002 | *cdtA- / cdtB- / tcdA+ / tcdB+* | 37.6 |
| 2 | 002 | *cdtA- / cdtB- / tcdA+ / tcdB+* | 30.5 |
| 3 | 002 | *cdtA- / cdtB- / tcdA+ / tcdB+* | 37.1 |
| 4 | 002 | *cdtA- / cdtB- / tcdA+ / tcdB+* | 35.9 |
| 5 | 005* | *cdtA- / cdtB- / tcdA+ / tcdB+* | 29.0 |
| 6 | 045 | *cdtA+ / cdtB+ / tcdA+ / tcdB+* | 36.4 |
| 7 | -- | *cdtA+ / cdtB+ / tcdA+ / tcdB+* | 30.0 |
| 8 | 078 | *cdtA+ / cdtB+ / tcdA+ / tcdB+* | 34.5 |
| 9 | 126 | *cdtA+ / cdtB+ / tcdA+ / tcdB+* | 36.6 |
| 10 | 078 | *cdtA+ / cdtB+ / tcdA+ / tcdB+* | 27.9 |
| 11 | 078 | *cdtA+ / cdtB+ / tcdA+ / tcdB+* | 28.8 |
| 12 | 078 | *cdtA+ / cdtB+ / tcdA+ / tcdB+* | 32.0 |
| 13 | 078 | *cdtA+ / cdtB+ / tcdA+ / tcdB+* | 32.9 |
| 14 | 078 | *cdtA+ / cdtB+ / tcdA+ / tcdB+* | 27.2 |
| 15 | 062* | *cdtA- / cdtB- / tcdA+ / tcdB+* | 33.5 |
| 16 | 015* | *cdtA- / cdtB- / tcdA+ / tcdB+* | 38.3 |
| 17 | 001 | insufficient material | 35.6 |
| 18 | 001* | *cdtA- / cdtB- / tcdA+ / tcdB+* | 29.2 |
| 19 | 001 | *cdtA- / cdtB- / tcdA+ / tcdB+* | 34.2 |
| 20 | 001 | *cdtA- / cdtB- / tcdA+ / tcdB+* | 33.1 |
| 21 | 001 | *cdtA- / cdtB- / tcdA+ / tcdB+* | 33.3 |
| 22 | 626 | *cdtA- / cdtB- / tcdA+ / tcdB+* | 31.4 |
| 23 | 037 | *cdtA- / cdtB- / tcdA+ / tcdB+* | 32.6 |
| 24 | 015 | *cdtA- / cdtB- / tcdA+ / tcdB+* | 35.7 |
| 25 | 626 | *cdtA- / cdtB- / tcdA+ / tcdB+* | 36.7 |
| 26 | 244* | *cdtA- / cdtB- / tcdA+ / tcdB+* | 35.1 |
| 27 | 011 | *cdtA- / cdtB- / tcdA+ / tcdB+* | 37.3 |
| 28 | 050 | *cdtA- / cdtB- / tcdA+ / tcdB+* | 29.3 |
| 29 | 258 | *cdtA- / cdtB- / tcdA+ / tcdB+* | 35.8 |
| 30 | 190 | *cdtA- / cdtB- / tcdA+ / tcdB+* | 36.7 |
| 31 | 258 | *cdtA- / cdtB- / tcdA+ / tcdB+* | 37.2 |
| 32 | 258 | *cdtA- / cdtB- / tcdA+ / tcdB+* | 30.9 |
| 33 | 190 | *cdtA- / cdtB+ / tcdA+ / tcdB+* | 30.8 |
| 34 | 216 | *cdtA- / cdtB- / tcdA+ / tcdB+* | 29.9 |
| 35 | 023 | *cdtA+ / cdtB+ / tcdA+ / tcdB+* | 29.6 |
| 36 | 026 | *cdtA- / cdtB- / tcdA+ / tcdB+* | 36.5 |
| 37 | 026 | *cdtA- / cdtB- / tcdA+ / tcdB+* | 31.5 |
| 38 | 026 | *cdtA- / cdtB- / tcdA+ / tcdB+* | 28.8 |
| 39 | 026 | *cdtA- / cdtB- / tcdA+ / tcdB+* | 35.3 |
| 40 | 026 | *cdtA- / cdtB- / tcdA+ / tcdB+* | 37.1 |
| 41 | 081 | *cdtA- / cdtB- / tcdA+ / tcdB+* | 40.0 |
| 42 | 029 | *cdtA- / cdtB- / tcdA+ / tcdB+* | 31.6 |
| 43 | 012 | *cdtA- / cdtB- / tcdA+ / tcdB+* | 37.7 |
| 44 | 012 | *cdtA- / cdtB- / tcdA+ / tcdB+* | 28.3 |
| 45 | 012 | *cdtA- / cdtB- / tcdA+ / tcdB+* | 31.7 |
| 46 | 014 | *cdtA- / cdtB- / tcdA+ / tcdB+* | 38.9 |
| 47 | 207 | *cdtA- / cdtB- / tcdA+ / tcdB+* | 38.9 |
| 48 | ? | *cdtA- / cdtB- / tcdA+ / tcdB+* | 32.5 |
| 49 | 014 | *cdtA- / cdtB- / tcdA+ / tcdB+* | 29.1 |
| 50 | 014 | *cdtA- / cdtB- / tcdA+ / tcdB+* | 32.7 |
| 51 | 014 | *cdtA- / cdtB- / tcdA+ / tcdB+* | 34.5 |
| 52 | 070 | *cdtA- / cdtB- / tcdA+ / tcdB+* | 31.9 |
| 53 | 017 | *cdtA- / cdtB- / tcdA+ / tcdB+* | 33.7 |
| 54 | 017 | *cdtA- / cdtB- / tcdA+ / tcdB+* | 36.7 |
| 55 | 017 | *cdtA- / cdtB- / tcdA+ / tcdB+* | 34.6 |
| 56 | 017 | *cdtA- / cdtB- / tcdA+ / tcdB+* | 35.3 |
| 57 | 017 | *cdtA- / cdtB- / tcdA+ / tcdB+* | 30.6 |
| 58 | 017 | *cdtA- / cdtB- / tcdA+ / tcdB+* | 30.9 |
| 59 | 017 | *cdtA- / cdtB- / tcdA+ / tcdB+* | 31.1 |
| 60 | 017 | *cdtA- / cdtB- / tcdA+ / tcdB+* | 30.1 |
| 61 | 017 | *cdtA- / cdtB- / tcdA+ / tcdB+* | 29.8 |
| 62 | 017 | *cdtA- / cdtB- / tcdA+ / tcdB+* | 31.6 |
| 63 | 265 | *cdtA- / cdtB- / tcdA+ / tcdB+* | 30.1 |
| 64 | 050 | *cdtA- / cdtB- / tcdA+ / tcdB+* | 28.5 |
| 65 | 027 | *cdtA+ / cdtB+ / tcdA+ / tcdB+* | 29.7 |

**Supplementary Table 2** – Details of all 65 fecal samples with positive qPCR for *C. difficile* toxin A and/or B genes and their 65 corresponding cultured strains. Samples are ordered and numbered according to their order on the X-axis in figure 2 (from left to right). All *C. difficile* fecal samples studied were also cultured and these strains were sent to the Dutch National Reference Laboratory for *C. difficile* at Leiden University Medical Center (LUMC) for conventional ribotyping using a standardized protocol (18). Toxin genes were detected in fecal samples by using toxin gene primers described by Persson et al (17). Cp values of *C. difficile* toxin A and/or B qPCR on fecal samples (the standard diagnostic test for *C. difficile* detection in our laboratory) indicates the *C. difficile* bacterial load in the studied fecal samples. *‘*’ = probable reference ribotype; ‘?’ = unknown reference ribotype; ‘-’ = no reference ribotype; tcdA = C. difficile toxin A; tcdB = C. difficile toxin B; cdtA = C. difficile binary toxin A; cdtB = C. difficile binary toxin B*
